# Supplementary material for: Preservation of co-expression defines the primary tissue fidelity of human neural organoids
Source: bioRxiv. 2023 Mar 31:2023.03.31.535112. Preprint. [Version 1] doi: 10.1101/2023.03.31.535112 (PMC10081321; doi:10.1101/2023.03.31.535112)
Supplement: Supplementary file 1 [file media-1.pdf]

Preservation of co-expression defines the primary tissue fidelity of human neural organoids

Jonathan M. Werner[1], Jesse Gillis[1,2]

[1]The Stanley Institute for Cognitive Genomics, Cold Spring Harbor Laboratory, Cold Spring Harbor, NY 11724, USA

[2]Physiology Department and Donnelly Centre for Cellular and Biomolecular Research, University of Toronto, Toronto, ON, Canada

Supplemental data

Supplemental Table 1

Table containing the study origin and download links for all primary tissue and organoid scRNA-seq datasets. The batch variable column details the meta-data used in determining batch. The region/protocol column details the sampled primary tissue brain regions or the organoid differentiation protocol.

Supplemental Table 2

Table containing our mapping between author provided annotations (Author annotations column) and our broad cell-type annotations (Class annotations column).

Supplemental Figure 1

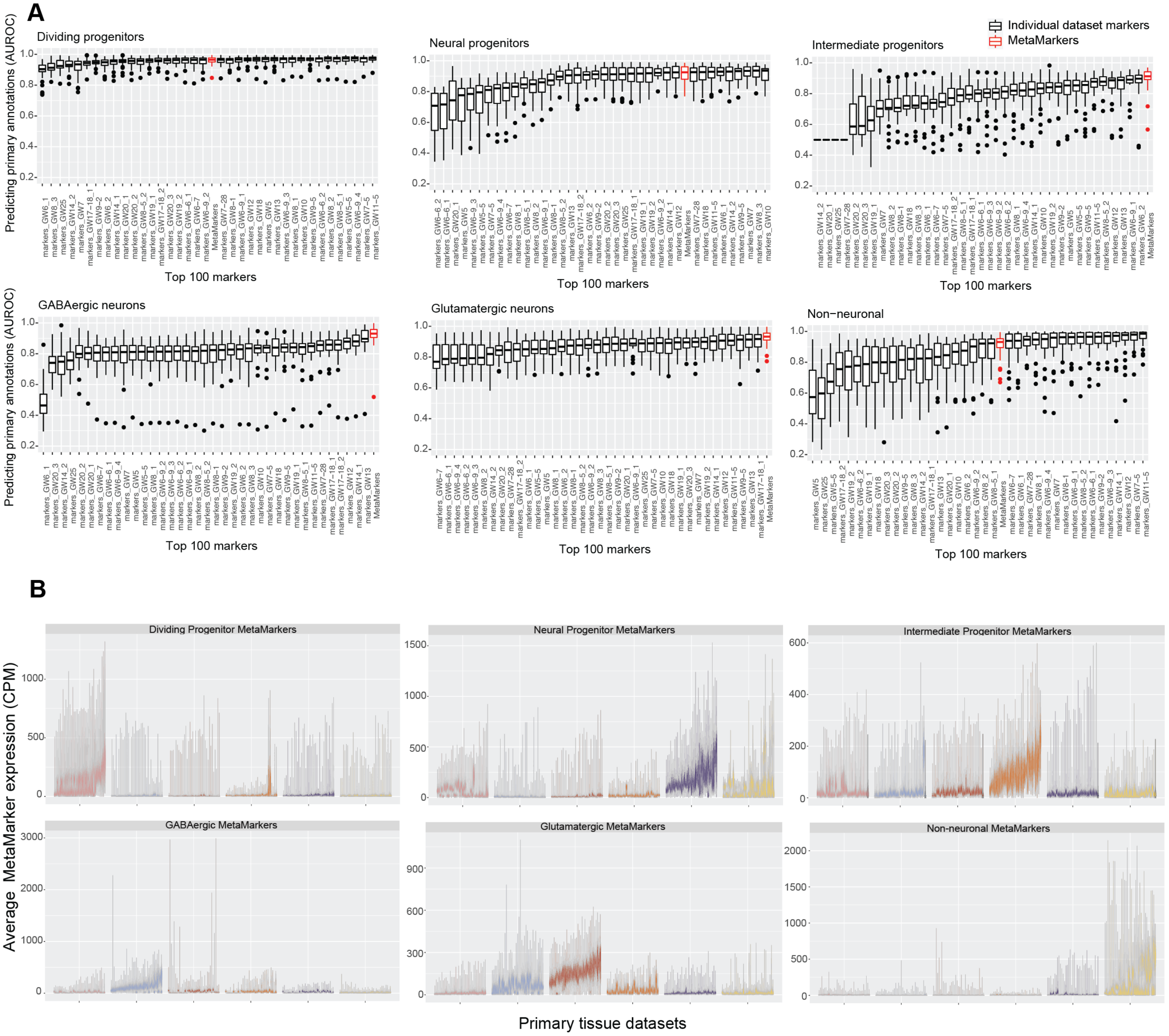

A MetaMarkers are consistent top performers in predicting primary tissue cell-type annotations. Boxplots of AUROCs for predicting cell-type annotations across all primary tissue datasets using the top 100 marker genes per individual primary tissue dataset compared to MetaMarkers (red). Datasets are ordered by their median performance, providing the rank distributions in Figure 2D.

B MetaMarkers exhibit cell-type specificity across all primary tissue datasets. Averaged distributions of gene expression for the top 100 MetaMarkers across all annotated primary tissue datasets with leave-one-out cross-validation. Figure 2E is the aggregate over these individual dataset distributions.

Supplemental Figure 2

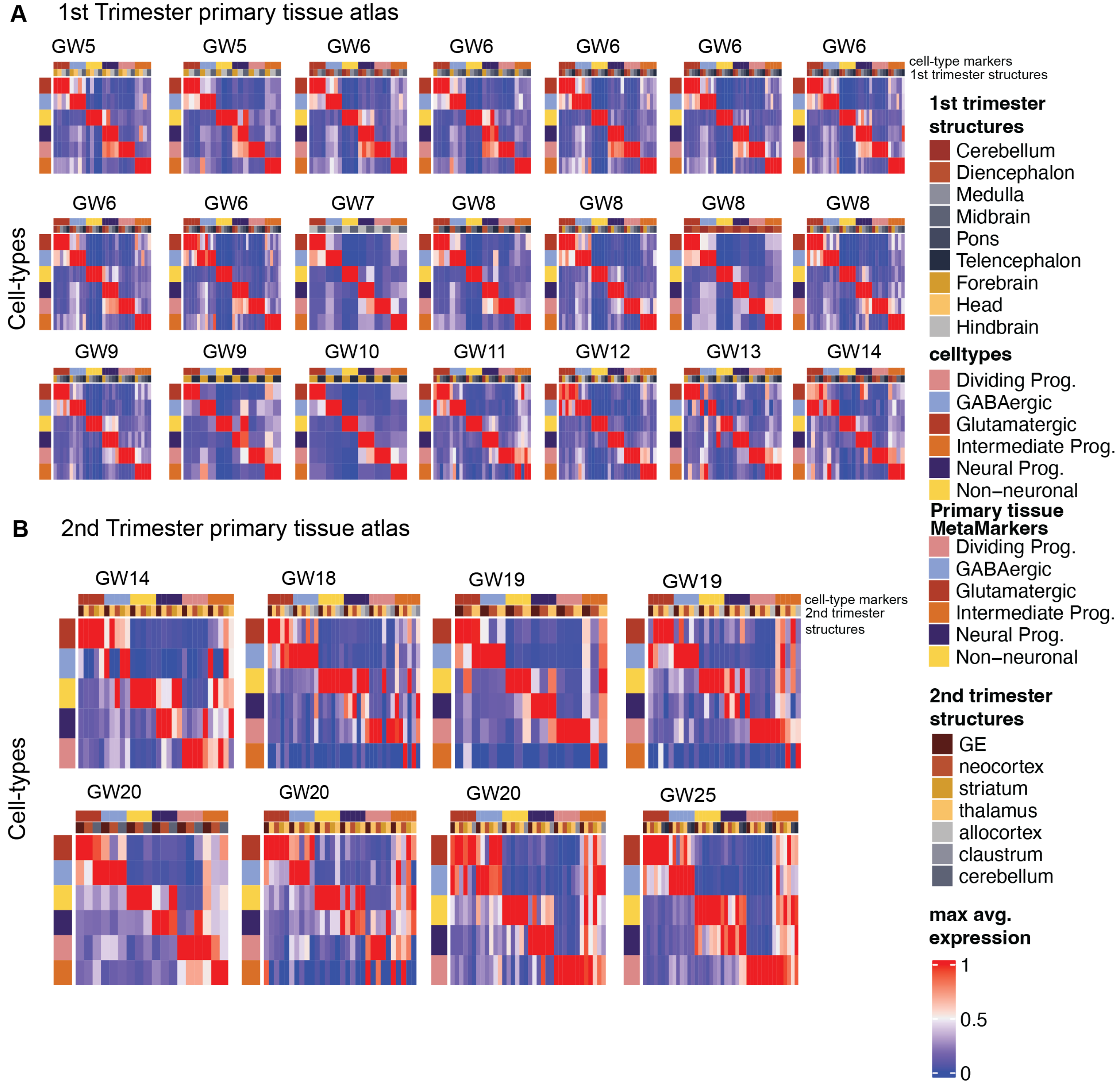

A MetaMarkers exhibit cross-regional cell-type specificity. Heatmaps of maximum normalized average MetaMarker expression for cell-types and brain regions of the first trimester annotated primary tissue atlas. Cell-types comprise the rows with MetaMarker gene expression for cells from each annotated brain region comprising the columns. Data is maximum normalized per region/column.

B MetaMarkers exhibit cross-regional cell-type specificity. Heatmaps of maximum normalized average MetaMarker expression for cell-types and brain regions of the second trimester annotated primary tissue atlas. Cell-types comprise the rows with MetaMarker gene expression for cells from each annotated brain region comprising the columns. Data is maximum normalized per region/column.

Supplemental Figure 3

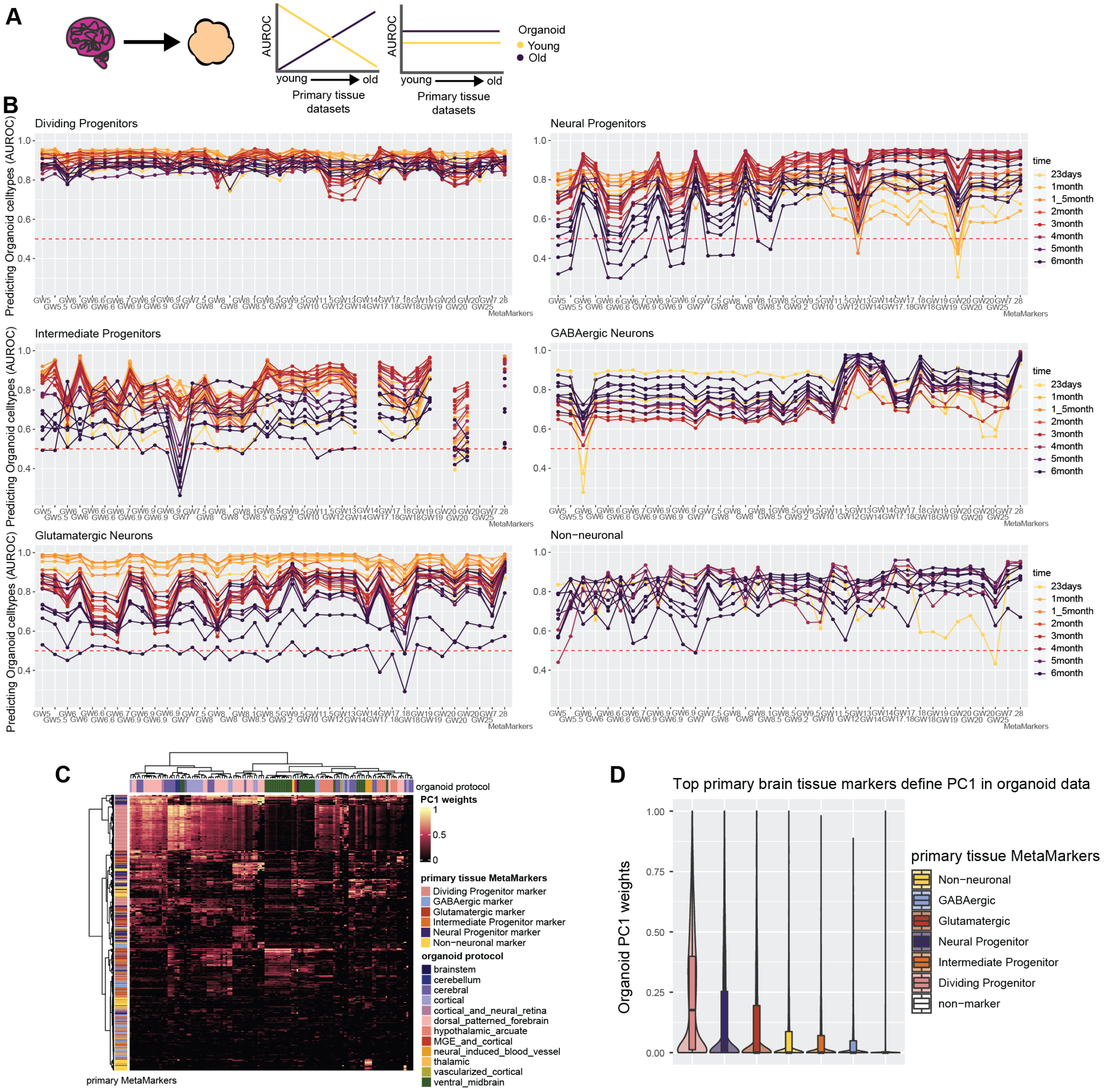

A Schematic showing two potential outcomes when comparing cell-type marker expression between primary tissue and organoid data on a temporal axis. There may be a temporal relationship, with younger organoids recapitulating younger primary tissue marker expression over older primary tissue marker expression and vice versa for older organoids, or there may be no temporal relationship.

B Broad primary tissue cell-type markers have consistent performance predicting organoid annotations independent of temporal variation. Line plots showing the cell-type prediction AUROCs using top 100 markers from individual primary tissue datasets for all organoid time points. Primary tissue datasets on the x-axis are ordered from youngest to oldest.

C Primary tissue MetaMarkers define the first organoid principal component. Heatmap of normalized eigenvalues for primary tissue MetaMarkers within the first principal component of each organoid dataset.
D MetaMarker gene-set distributions of normalized PC1 eigenvalues across all organoid datasets.

Supplemental Figure 4

A

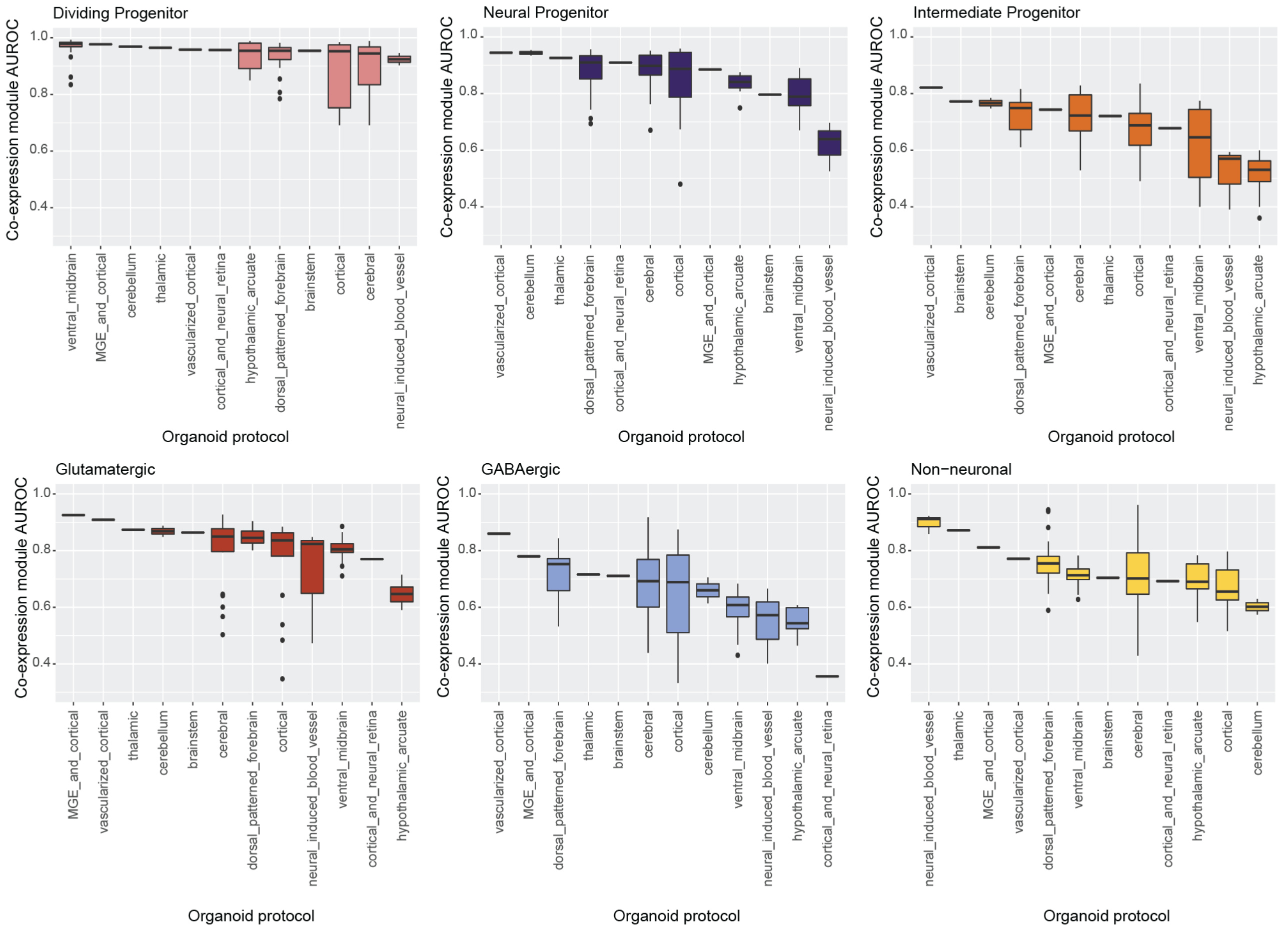

A Organoids vary by protocol type for their primary tissue cell-type co-expression module scores. Boxplot distributions of co-expression module scores for the primary tissue MetaMarkers computed from organoid co-expression networks. Scores for organoid networks are grouped by organoid protocol type and ordered by their median score.

Supplemental Figure 5

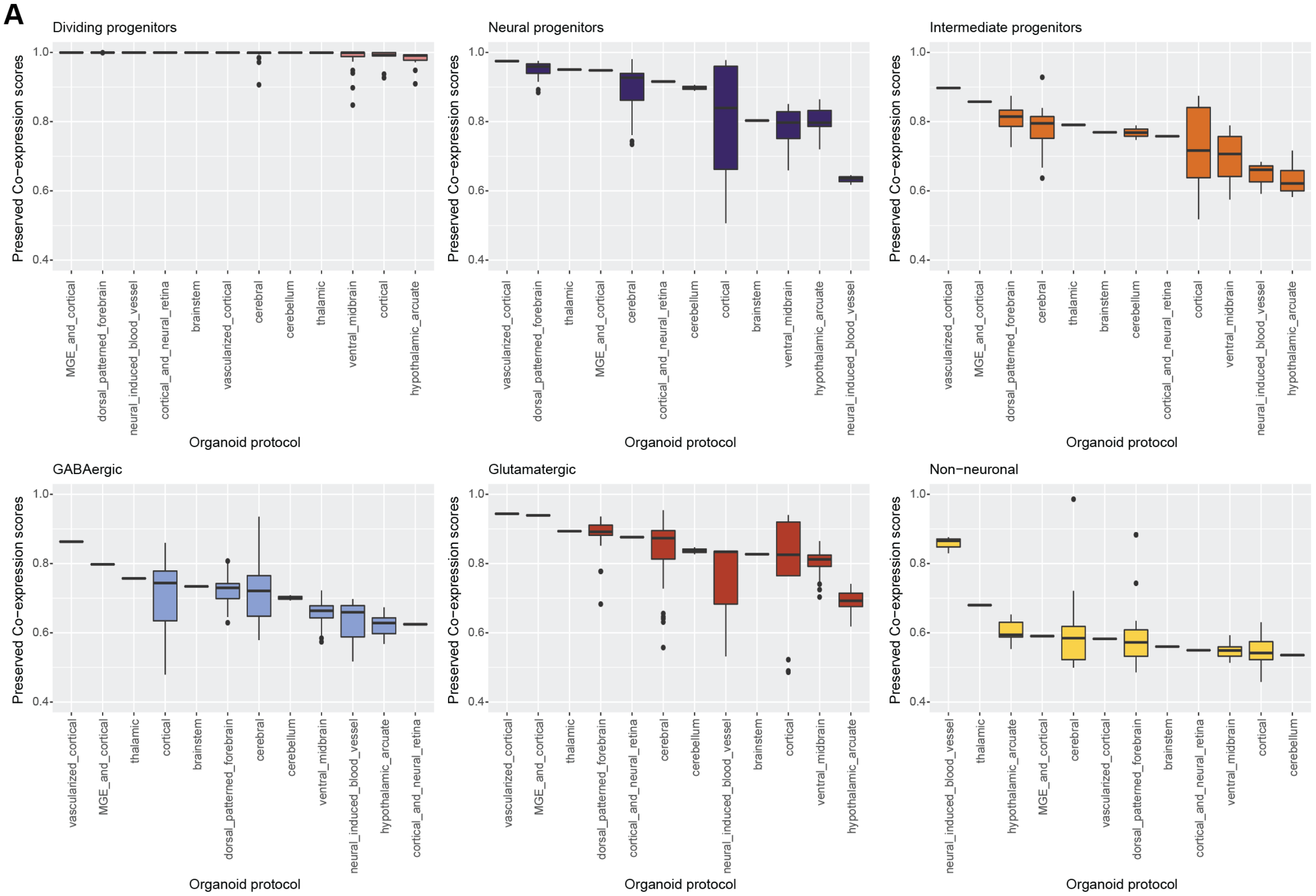

A Organoids vary by protocol type for their primary tissue cell-type preserved co-expression scores. Boxplot distributions of preserved co-expression scores for the primary tissue MetaMarkers computed from organoid co-expression networks. Scores for organoid networks are grouped by organoid protocol type and ordered by their median score.

Supplemental Figure 6

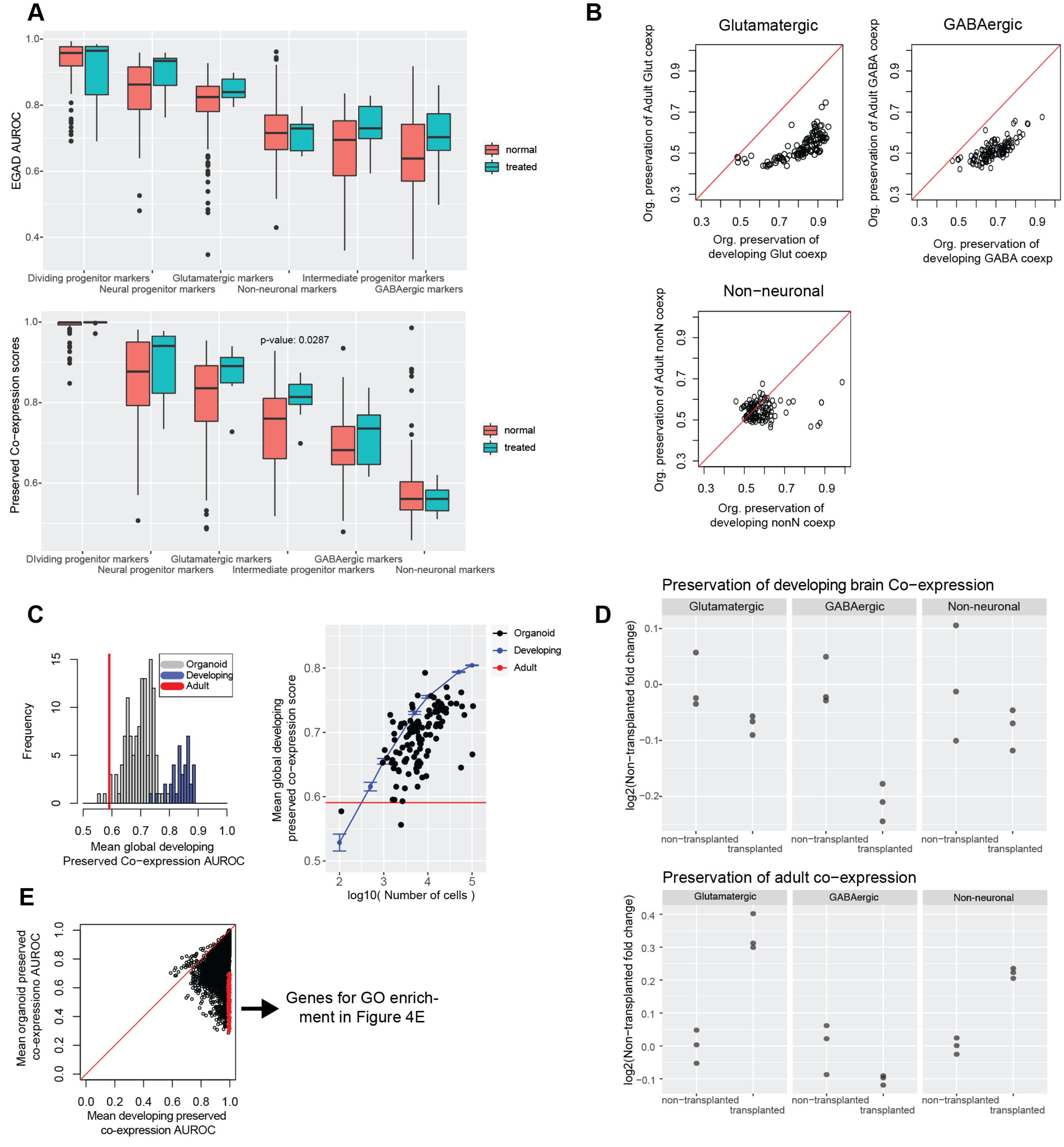

A Normal and treated organoids exhibit no differences in their recapitulation of primary tissue co-expression. Boxplots comparing either the co-expression module scores or preserved co-expression scores by cell-type between normal and treated organoids.

B Organoids preserve developing neuronal co-expression over adult co-expression. Scatterplots showing the preserved co-expression scores of either the top 100 developing brain MetaMarkers (x-axis) or the top 100 adult MetaMarkers (y-axis).

C Organoids lie between adult and developing brain data for global preservation of developing brain co-expression. Distributions of average preserved developing brain co-expression AUROCs across all genes for organoid and developing brain networks. The redline shows the performance of the adult co-expression network. The scatterplot plots the data in the histogram (y-axis) against the number of cells in each organoid dataset (x-axis). The blue line shows performance for a cell down-sampled developing brain dataset, with points representing the average performance over 10 random samples and the error bars showing ± 1 standard deviation.
D Transplanted organoids preserve adult co-expression over developing brain co-expression. Points represent the log2-fold change over the mean performance of the non-transplanted organoids for preserved co-expression scores.
E Organoids globally have low preserved developing brain co-expression of individual genes across the genome. Points show the average preserved developing brain co-expression AUROC of individual genes, comparing the average across developing brain networks (x-axis) against the average across organoid networks (y-axis). The points colored in red are genes with developing brain scores >= 0.99 and organoid scores < 0.70.

Supplemental Figure 7

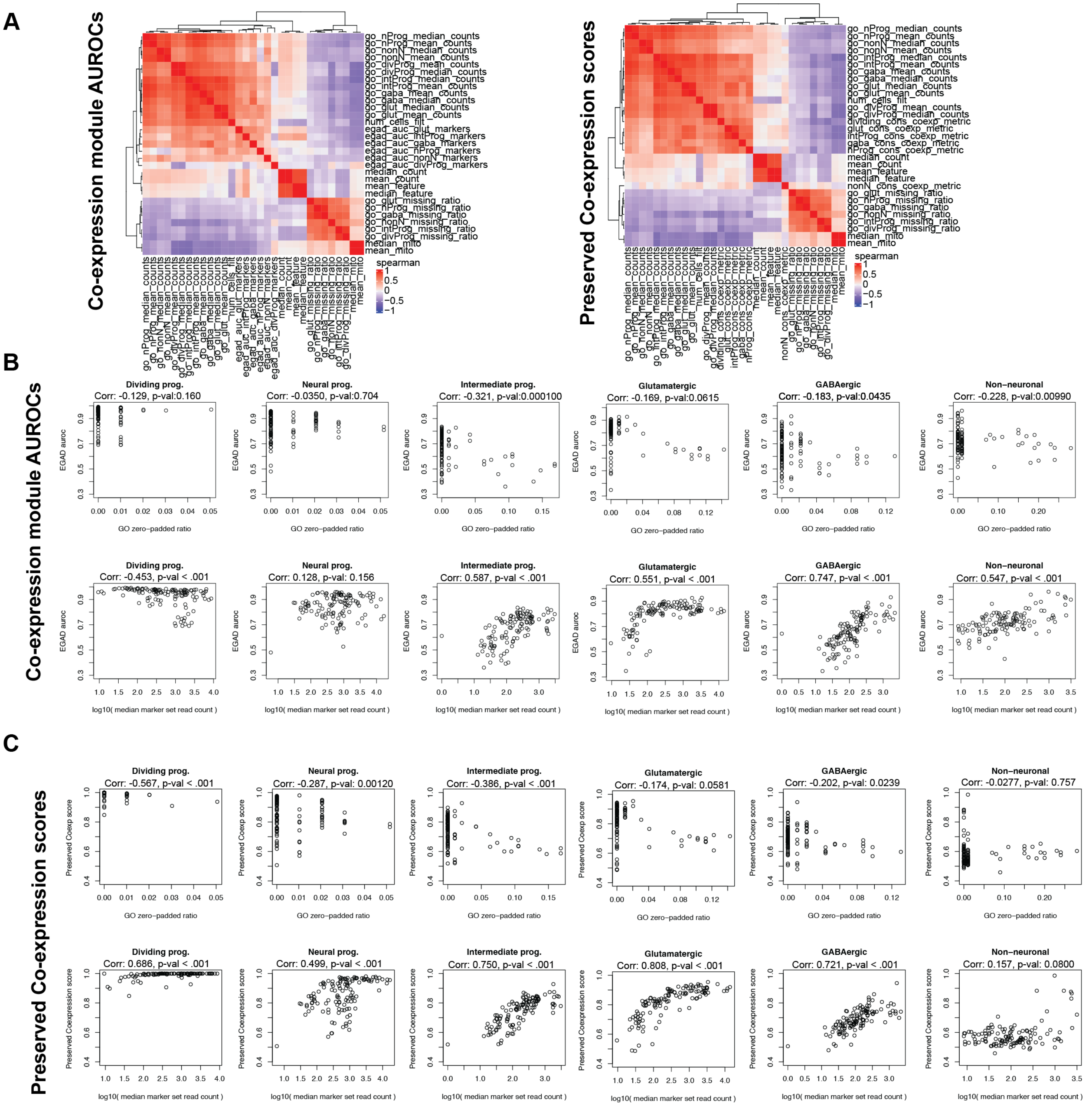

A Marker set expression and cell number are strongly correlated with co-expression performance across organoid datasets. Heatmaps of spearman correlations between either co-expression module scores or preserved co-expression scores and various technical features of each network/dataset, like marker set expression, dataset sequencing depth, number of cells in each dataset, and the zero-padding ratio of each marker set.

B Scatterplots of either the zero-padded ratio (top row) or marker set expression (bottom row) against the co-expression module scores for each cell-type across the organoid datasets.

C Scatterplots of either the zero-padded ratio (top row) or marker set expression (bottom row) against the preserved co-expression scores for each cell-type across the organoid datasets.

References (Supp. Table 1)

1. Polioudakis, D. *et al.* A Single-Cell Transcriptomic Atlas of Human Neocortical Development during Mid-gestation. *Neuron* **103**, 785-801.e8 (2019).

2. Fan, X. *et al.* Single-cell transcriptome analysis reveals cell lineage specification in temporal-spatial patterns in human cortical development. *Science Advances* **6**, eaaz2978 (2020).

3. Bhaduri, A. *et al.* An atlas of cortical arealization identifies dynamic molecular signatures. *Nature* **598**, 200–204 (2021).

4. Braun, E. *et al.* Comprehensive cell atlas of the first-trimester developing human brain. 2022.10.24.513487 Preprint at https://doi.org/10.1101/2022.10.24.513487 (2022).

5. Shi, Y. *et al.* Mouse and human share conserved transcriptional programs for interneuron development. *Science* **374**, eabj6641 (2021).

6. Zhou, X. *et al.* Deciphering the spatial-temporal transcriptional landscape of human hypothalamus development. *Cell Stem Cell* **29**, 328-343.e5 (2022).

7. Yu, Y. *et al.* Interneuron origin and molecular diversity in the human fetal brain. *Nat Neurosci* **24**, 1745–1756 (2021).

8. Trevino, A. E. *et al.* Chromatin and gene-regulatory dynamics of the developing human cerebral cortex at single-cell resolution. *Cell* **184**, 5053-5069.e23 (2021).

9. Jorstad, N. L. *et al. Comparative transcriptomics reveals human-specific cortical features*. http://biorxiv.org/lookup/doi/10.1101/2022.09.19.508480 (2022) doi:10.1101/2022.09.19.508480.

10. Uzquiano, A. *et al.* Proper acquisition of cell class identity in organoids allows definition of fate specification programs of the human cerebral cortex. *Cell* **185**, 3770-3788.e27 (2022).

11. Fiddes, I. T. *et al.* Human-Specific NOTCH2NL Genes Affect Notch Signaling and Cortical Neurogenesis. *Cell* **173**, 1356-1369.e22 (2018).

12. Field, A. R. *et al.* Structurally Conserved Primate LncRNAs Are Transiently Expressed during Human Cortical Differentiation and Influence Cell-Type-Specific Genes. *Stem Cell Reports* **12**, 245–257 (2019).

13. Xiang, Y. *et al.* Fusion of Regionally Specified hPSC-Derived Organoids Models Human Brain Development and Interneuron Migration. *Cell Stem Cell* **21**, 383-398.e7 (2017).

14. Khan, T. A. *et al.* Neuronal defects in a human cellular model of 22q11.2 deletion syndrome. *Nat Med* **26**, 1888–1898 (2020).

15. Nayler, S., Agarwal, D., Curion, F., Bowden, R. & Becker, E. B. E. High-resolution transcriptional landscape of xeno-free human induced pluripotent stem cell-derived cerebellar organoids. *Sci Rep* **11**, 12959 (2021).

16. Fair, S. R. *et al.* Electrophysiological Maturation of Cerebral Organoids Correlates with Dynamic Morphological and Cellular Development. *Stem Cell Reports* **15**, 855–868 (2020).

17. Parisian, A. D. *et al.* SMARCB1 loss interacts with neuronal differentiation state to block maturation and impact cell stability. *Genes Dev* **34**, 1316–1329 (2020).

18. Chen, X. *et al.* Modeling Sporadic Alzheimer's Disease in Human Brain Organoids under Serum Exposure. *Adv Sci (Weinh)* **8**, 2101462 (2021).

19. Dailamy, A. *et al.* Programmatic introduction of parenchymal cell types into blood vessel organoids. *Stem Cell Reports* **16**, 2432–2441 (2021).

20. Banfi, F. *et al.* SETBP1 accumulation induces P53 inhibition and genotoxic stress in neural progenitors underlying neurodegeneration in Schinzel-Giedion syndrome. *Nat Commun* **12**, 4050 (2021).

21. Popova, G. *et al.* Human microglia states are conserved across experimental models and regulate neural stem cell responses in chimeric organoids. *Cell Stem Cell* **28**, 2153-2166.e6 (2021).

22. Suong, D. N. A. *et al.* Induction of inverted morphology in brain organoids by vertical-mixing bioreactors. *Commun Biol* **4**, 1–13 (2021).

23. Xiang, Y. *et al.* hESC-Derived Thalamic Organoids Form Reciprocal Projections When Fused with Cortical Organoids. *Cell Stem Cell* **24**, 487-497.e7 (2019).

24. Kronenberg, Z. N. *et al.* High-resolution comparative analysis of great ape genomes. *Science* **360**, eaar6343 (2018).

25. Pollen, A. A. *et al.* Establishing Cerebral Organoids as Models of Human-Specific Brain Evolution. *Cell* **176**, 743-756.e17 (2019).

26. Velasco, S. *et al.* Individual brain organoids reproducibly form cell diversity of the human cerebral cortex. *Nature* **570**, 523–527 (2019).

27. Shi, Y. *et al.* Vascularized human cortical organoids (vOrganoids) model cortical development in vivo. *PLOS Biology* **18**, e3000705 (2020).

28. Qian, X. *et al.* Sliced Human Cortical Organoids for Modeling Distinct Cortical Layer Formation. *Cell Stem Cell* **26**, 766-781.e9 (2020).

29. Eura, N. *et al.* Brainstem Organoids From Human Pluripotent Stem Cells. *Frontiers in Neuroscience* **14**, (2020).

30. Huang, W.-K. *et al.* Generation of hypothalamic arcuate organoids from human induced pluripotent stem cells. *Cell Stem Cell* **28**, 1657-1670.e10 (2021).

31. Fiorenzano, A. *et al.* Single-cell transcriptomics captures features of human midbrain development and dopamine neuron diversity in brain organoids. *Nat Commun* **12**, 7302 (2021).

32. Fernando, M. *et al.* Differentiation of brain and retinal organoids from confluent cultures of pluripotent stem cells connected by nerve-like axonal projections of optic origin. *Stem Cell Reports* **17**, 1476–1492 (2022).

33. Szebényi, K. *et al.* Human ALS/FTD brain organoid slice cultures display distinct early astrocyte and targetable neuronal pathology. *Nat Neurosci* **24**, 1542–1554 (2021).

34. Quadrato, G. *et al.* Cell diversity and network dynamics in photosensitive human brain organoids. *Nature* **545**, 48–53 (2017).

35. Revah, O. *et al.* Maturation and circuit integration of transplanted human cortical organoids. *Nature* **610**, 319–326 (2022).
